# Shear Bond Strength and Mode of Failure of Polypropylene Fibers in Orthodontic Flash-Free Adhesive

**DOI:** 10.3390/polym14194167

**Published:** 2022-10-04

**Authors:** Kitiporn Chaimaungchuen, Apiwat Riddhabhaya, Nattisa Niyomtham, Irin Sirisoontorn

**Affiliations:** 1 Department of Clinical Dentistry, Walailak University International College of Dentistry (WUICD), 87 Ranong 2 Road, Dusit, Bangkok 10300, Thailand; 2 Department of Oral Health Science, Walailak University International College of Dentistry (WUICD), 87 Ranong 2 Road, Dusit, Bangkok 10300, Thailand

**Keywords:** ultrasonic scaler, shear bond strength, orthodontics, flash-free adhesive, dentistry, ARI score

## Abstract

The purpose of this research is to evaluate the effects of magnetostrictive and piezoelectric scalers on the shear bond strength (SBS), failure mode of the polypropylene fiber adhesive brackets, and the load of both scaler tips. The adhesive precoated (APC) Flash-Free brackets were placed on the buccal surfaces of sixty maxillary first premolars, which were divided into three equal groups of 20 specimens each, following the control group (no scaling), the magnetostrictive group, and the piezoelectric group. All specimens were measured for SBS value by using a universal testing machine at a crosshead speed of 0.75 mm/minute. The mode of failure was examined under a×10 magnification light microscope digital camera and scores for the adhesive remnant index (ARI) were recorded and measured load between the magnetostrictive and piezoelectric groups. For statistical analysis, ANOVA and multiple comparison, as well as unpaired *t*-test and chi-square tests, at the 0.05 significance level were used. The results showed that the average SBS value of the control group was greater than that of the magnetostrictive group and the piezoelectric group. However, the SBS was not significantly influenced by ultrasonic instruments (*p* > 0.05). The ARI score and load showed no significant differences among the groups (*p* > 0.05). In conclusion, the SBS of the APC Flash-Free bracket wasn’t affected by using ultrasonic instrumentation around the base of the bracket.

## 1. Introduction

Orthodontics has always represented a successful and appropriate treatment to modify tooth position for functional and esthetic purposes. A successful treatment is achieved through the use of fixed buccal or lingual brackets or through aligners [1,2,3].

**Figure 1:**
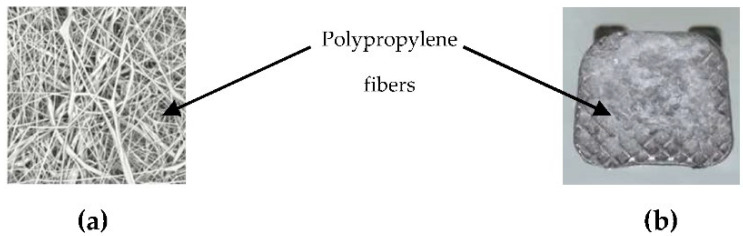
(**a**) The nonwoven polypropylene fibers used in the APC^TM^ Flash-Free adhesive consists of randomly oriented, entangled fibers (3M^TM^ APC^TM^ Flash-Free Adhesive, Technical Overview); (**b**) The image from the light microscope digital camera with ×10 magnification (EP50 Olympus, Tokyo, Japan).

Adhesive Precoated (APC) brackets were first produced by 3M Unitek in 1991. Since then, the company has introduced the APC II and APC PLUS bonding technologies, both of which are regarded as significant upgrades. Then, the specified adhesives were modified by substituting a low-viscosity resin for a compomer (an APC Flash-Free adhesive). The base is a low-viscosity resin that has been soaked in a nonwoven polypropylene fiber material (Figure 1). Therefore, by skipping the step of clearing up the flash surrounding the bracket, the bonding process is quickened [4].

Dental hygiene is an important factor that the patient can control during orthodontic treatment, and it can affect the quality and timing of treatment [5]. Gingival tissue commonly exhibits an inflammatory response during orthodontic treatment with fixed appliances. The retentive areas in and around parts of orthodontic appliances attached to the teeth are the primary cause of increased dental plaque deposition and inflammatory response [6]. Plaque accumulation is facilitated on the cervical surface of brackets, below the leveling arch, and is exacerbated by the patients’ difficulty cleaning these sites [7]. As a result, orthodontic patients’ oral hygiene guidelines also include verbal knowledge as well as professional treatments using ultrasonic scalers and rotating brushes [8].

There are two types of ultrasonic scaler used in dentistry today: magnetostrictive and piezoelectric. Their action mechanisms are distinct [9]. Magnetostrictive units operate between 18 kHz and 45 kHz by applying an AC electromagnetic field to a stack of nickel strips, which then contract along their length by producing longitudinal vibrations. These vibrations move from the stack across a body connection to a tip, and the movement of the tip is elliptical. The piezoelectric units operate in the 25–50 kHz range; the AC electrical current applied across a ceramic or quartz disk causes it to expand, then contract, producing longitudinal vibrations that move at the attached end; tip movement is primarily linear in direction [10].

Previous studies have shown that a piezoelectric scaler influences and reduces the shear bond strength (SBS) of the APC ll and conventional orthodontic brackets, indicating a higher probability of bracket bond failure after oral hygiene procedures performed by professionals [11,12]. Damaged brackets can make orthodontic treatment take longer, more expensive, and take more time in the dentist’s chair, which can be inconvenient for both the patient and the dentist [13].

However, the impact of ultrasonic instrumentation on the shear bond strength between magnetostrictive and piezoelectric types has not been investigated. The goal of this research was to find out how magnetostrictive and piezoelectric scalers affect the shear bond strength and failure mode of polypropylene fiber adhesive brackets as well as the load on both scaler tips. The first null hypothesis was that there is no significant difference in the shear bond strength of APC Flash-Free adhesive after using different ultrasonic instrumentations. The second null hypothesis was that there are no differences in load between magnetostrictive and piezoelectric scalers.

## 2. Materials and Methods

### 2.1. Specimen Preparation

Sixty maxillary first premolars were extracted for orthodontic reasons. Teeth were stored in a 0.1% thymol solution after extraction, then washed to eliminate contamination and kept in distilled water in the fridge for 1–6 months [14]. The teeth had to be sound and not have visible cracks, labial restorations, caries, white spots or hypoplasia under×4 magnification light microscope digital camera (EP50 Olympus, Tokyo, Japan). The use of human teeth in research has been approved by the Research Institute for Health Science at Walailak University (project no. WU-EC-DE-1-416-63).

The teeth were cut off below the cementoenamel junction (CEJ) by 2 mm with a diamond disc (Intensiv^TM^, Zurich, Switzerland) and micromotor (Marathon-3 champion, SEAYANG, Daegu, Korea). After that, the teeth were placed on pink wax (2 mm thickness) that covered the glass lab. The teeth were then covered with a polyvinyl chloride mold and fixed with self-curing acrylic resin (UNIFAST™ Trad, GC America Inc, Alsip, IL, USA). Each tooth was positioned horizontally so that the labial surface paralleled the floor.

### 2.2. Bonding Procedure

After being cleansed with rubbing alcohol, the facial surface of each premolar was wax-free. Following a fluoride-free pumice scrub, the surface was rinsed and dried with oil-free compressed air. The manufacturer’s instructions were carefully followed for etching with 37% phosphoric acid gel (Scotchbond™; 3M Unitek, Irwindale, CA, USA) for 15 s, followed by thorough washing and drying until a characteristic frosty white etched area was observed. Afterwards, a thin uniform coat of primer (Transbond™ XT; 3M Unitek) was applied by using a microbrush, followed by a gentle oil- and moisture-free air burst to dry.

**Figure 2:**
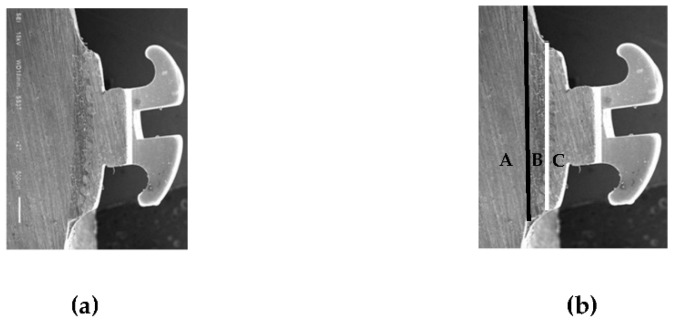
(**a**) Cross-sectional SEM image of the teeth with metallic bracket (original magnification ×27, bar represents 500 μm); (**b**) A, enamel layer; B, adhesive layer; C, bracket base layer; black line, enamel–adhesive interface; white line, bracket base–adhesive interface.

In the experiment described in the report by Bishara et al. [15], polypropylene fiber adhesive brackets (APCTM Flash-Free Adhesive brackets; Victory Series; 3M Unitek) were placed on the teeth, their actual positions (on the middle one third of the buccal surface) were adapted, and they were loaded with a 300 g force by the loading device. The adhesive was cured by using a LED light curing unit (Elipar^TM^ S10; 3M Unitek) for 20 s (10 s medially and 10 s distally), according to the manufacturer’s instructions (Figure 2). Before use, it was ensured that the light intensity was at 100%. All samples were then kept in water at 37 °C for 24 h before scaling [14].

### 2.3. Ultrasonic Instrumentation

The specimens were divided into three groups of 20 at random: control (no scaling), magnetostrictive (MS), and piezoelectric (PE).

A magnetostrictive scaler (Pentazon^®^ VRT-07, Pentamed, Bangkok, Thailand) was used with the original prophy insert tip (Hu-friedy, Chicago, IL, USA). The instrument was operated according to the manufacturer’s instructions under profuse rinsing with water spray at full power.

A piezoelectric scaler (Newtron^®^ P5 XS B.LED, Satelec, Acteon, France) was used at power level 13 (recommended by the manufacturer for scale removal) with a 0° scaler tip (Universal tip, #1, Satelec, Acteon, France) and water from the tank as a coolant.

**Figure 3:**
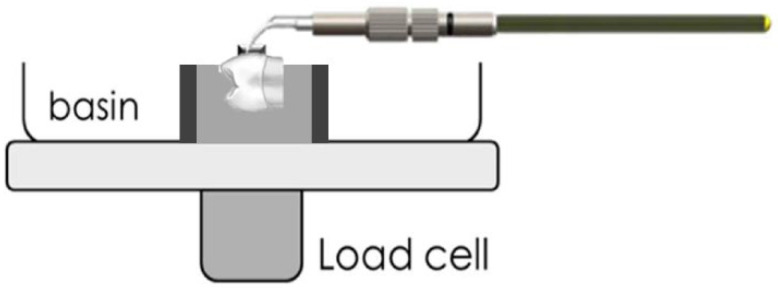
Illustration of applied ultrasonic instrumentation.

Before using an ultrasonic scaler, all specimens were tested in a load cell. As illustrated in Figure 3, each sample received 15 s of ultrasonic instrumentation on either side of the bracket (total time: 1 min) [12]. All of these treatments were carried out by a single experienced and trained operator.

### 2.4. Data Collection

#### 2.4.1. Shear Bond Strength

All specimens were kept in distilled water at 37 °C for 24 h after scaling. Each acrylic block was tested using universal testing equipment (Model 5566; Instron^®^ Co., Norwood, MA, USA) with the bracket base parallel to the applied force. The shear bond strength was next measured at a crosshead speed of 0.75 mm/min [14]. The measured values were then divided by the surface area of the bracket’s bottom and converted to MPa units. Image analysis tools (the ImageJ program from the NIH in the US) were used to measure the base area of the bracket (10.04 mm^2^).

#### 2.4.2. Mode of Failure

**Figure 4:**
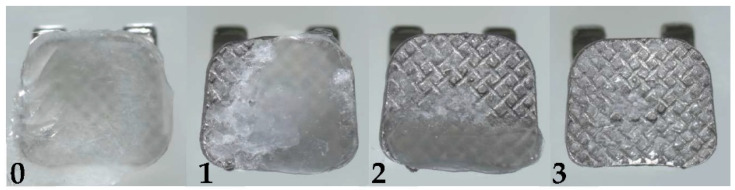
ARI, adhesive remnant index; 0, all adhesive remains on the bracket base; 1, ≥50% of the adhesive remains on the bracket base; 2, <50% of the adhesive remains on the bracket base; and 3, no adhesive remains on the bracket base.

The brackets were examined using a digital camera equipped with an X10 magnification light microscope (EP50 Olympus, Tokyo, Japan). The adhesive residual following bracket removal was analyzed using the Adhesive Remnant Index (ARI) and scored based on the amount of adhesive material left on the bracket base and expressed as a percentage of the bracket base area. The purpose of the ARI was to classify the locations and types of bonding failure between the tooth surface, adhesive, and bracket. The ARI was recorded on a scale from 0 to 3. As shown in Figure 4, 0 indicates that all adhesive remains on the bracket base; 1 indicates that more than half the adhesive remains on the bracket base; 2 indicates that less than half the adhesive remains on the bracket base; and 3 indicates that no adhesive remains on the bracket base [16].

The evaluation was carried out by an examiner who had received software training and was blind to the groups. The same evaluator repeated the evaluations a week later.

#### 2.4.3. Load

On each specimen, ultrasonic instrumentation was performed for 15 s/side of the bracket; mesial, distal, occlusal, and gingival (total time:1 min) [12]. During scaling, the device will record the load results on a universal testing machine (Model 5566; Instron^®^ Co., Norwood, MA, USA)

### 2.5. Statistical Analysis

All statistical analyses were performed using the Statistical Package for Social Sciences (SPSS) for Mac (28.0.1.1; SPSS, Chicago, IL, USA). Descriptive statistics (mean, standard deviation, minimum, and maximum values) were calculated for all groups. The Shapiro-Wilk test was used to determine the data’s normality. For individual within-group comparisons, one-way ANOVA was used, followed by the Bonferroni test of bond strength values. The unpaired *t*-test was used to determine significant differences in the load values. The chi-square test was used to determine the significant differences in the distribution of ARI scores between the various groups. The level of significance was set at 0.05.

## 3. Results

### 3.1. Shear Bond Strength (SBS)

The Shear Bond Strength Descriptive Statistics (MPa) of the three groups are presented in Table 1. The control group’s mean SBS value was 9.65 ± 0.44 Mpa, which was higher than that of the PE group (9.45 ± 0.67 MPa) and the MS group (9.54 ± 0.51 MPa). However, the SBS was not significantly different among groups (*p* > 0.05).

### 3.2. Mode of Failure

**Figure 5:**
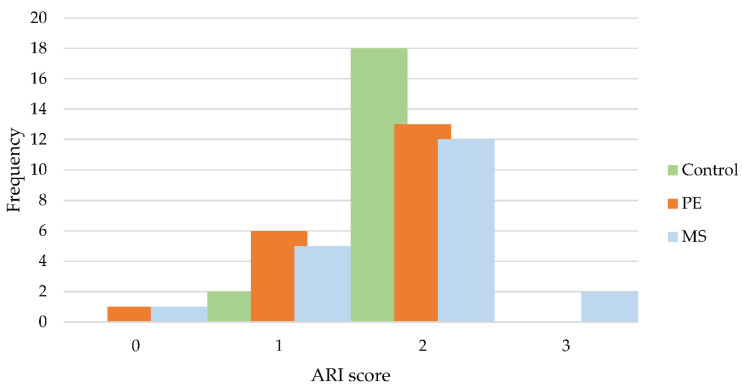
Distribution of adhesive remnant index (ARI) scores.

Table 2 and Figure 5 show the distribution of ARI scores. Most of the brackets in all groups showed an ARI score of 2. The most prevalent type of failure was at the enamel-adhesive level, with less than 50% of the adhesive remaining on the bracket base. On the other hand, the MS group demonstrated bond failure occurring at the adhesive-bracket level (ARI score 3). The chi-squared test showed no significant differences among the frequency distributions of various groups (*p* > 0.05).

### 3.3. Load

Descriptive load (*n*) statistics for all three groups are presented in Table 3. After using ultrasonic scalers around the bracket bases, both groups provided a similar load. There was no significant difference from the unpaired *t*-test (*p* > 0.05).

## 4. Discussion

### 4.1. Shear Bond Strength

Patients undergoing fixed orthodontic treatment must maintain adequate oral hygiene to reduce the risks of periodontal disease and enamel demineralization [17]. Typically, ultrasonic instrumentation is employed around the bracket base, which is a high-risk location for plaque accumulation [18]. This is the first study to look at the effects of magnetostrictive and piezoelectric ultrasonic devices on the SBS and failure mode of metallic brackets. Because magnetostrictive and piezoelectric ultrasonic instruments have various scaler tip oscillations and operating frequencies, dentists commonly use them for professional mouth cleansing operations on patients with fixed orthodontic appliances without considering their potential impacts. As a result, we sought to investigate and compare the effects of ultrasonic instruments on the SBS of metallic brackets, which had not before been studied.

In a previous study, the SBS of metallic orthodontic brackets with an ultrasonic instrument was examined to determine the impact of a piezoelectric device with various scaler-tip angulations (0°, 45°). They discovered that the scaler tip angulation has no effect on the SBS, but the piezoelectric device minimizes the SBS [11]. In our study, we found that using two different types of ultrasonic instruments has no statistically significant effect on the SBS. The first null hypothesis, which was accepted (*p* > 0.05), showed no significant difference in the shear bond strength of APC Flash-Free adhesive after using different ultrasonic instrumentations. However, in both scaler groups, average SBS values of APC Flash-Free brackets remained over 6 MPa, which was considered adequate for orthodontic purposes [19].

In 2017, Scribante et al. [12] found that ultrasonic appliances with a conventional lower bracket base area (<10 mm^2^) had significantly lower SBS values. Conversely, ultrasonic instrumentation had no effect on appliances with a larger base area (>10 mm^2^) [12]. All of the groups in our study used the polypropylene fiber adhesive brackets (APC^TM^; Flash-Free adhesive brackets; Victory Series; 3M Unitek), which have a bracket base area of 10.04 mm^2^. Furthermore, the advantages of APC include constant adhesive quality and amount, quicker cleanup after bonding, reduced waste during bonding, enhanced assessment, and better inventory control [20].

The force applied to the bracket during bonding influences the SBS. SBS increased along with the force. However, this trend decreased as it gained bonding forces, and no change in the shear bond occurred above 400 g [21]. Therefore, we used 300 g in this study, which was also in accordance with the experimental setup in the report of Bishara et al. [15].

Frictional heating can happen when the high-frequency oscillating tip comes into contact with the tooth’s surface [22]. Sato and Wakabayashi’s study showed that the temperature of the enamel surface increased when they used an ultrasonic scaler reposition bracket and went up even further when they did not use air cooling [23]. In this study, both of the ultrasonic scalers worked with a constant supply of water flowing over the tip as a coolant. This was done to minimize the amount of heat generated by friction.

Moreover, other types of scalers and the SBS of orthodontic brackets have been investigated. Oduncuoğlu et al. reported that sonic and piezoelectric ultrasonic instruments lowered the binding strength of metallic orthodontic brackets tested in vitro. The SBS was more impacted by instrumentation around the bracket base than the lingual surface. When applied around the bracket base, ultrasonic instruments demonstrated lower SBS than sonic instruments. Therefore, they recommended that instrumentation be done cautiously, particularly around the bracket base, as the application of sonic and ultrasonic periodontal instrumentation around the orthodontic metallic bracket base decreased bracket SBS [24]. 

Hatipoglu S. and Paksoy T. [13] reported in 2022 that using multiple ultrasonic and vector ultrasonic instruments reduced the SBS of conventional metallic brackets. Although vector ultrasound devices demonstrated higher values than ultrasound devices, there was no statistically significant difference between them. Both procedures can be used as multiple cleaning aids for patients receiving fixed orthodontic treatment with caution. However, many things could affect the SBS of orthodontic brackets in in vitro studies, like the material, the teeth, and other minor things [25]. In fact, many variables could have a significant influence on bond strength, such as adhesive type [26], surface contamination [27], and direct or enamel pretreatment [28]. These factors should be evaluated in future in vitro and clinical studies.

### 4.2. Mode of Failure

The finding that the groups’ ARI scores were similar shows that the mode of bracket bond failure was unaffected by the type of ultrasonic instrumentation. Most of the adhesive was still on the enamel in all of the groups, showing a low risk of enamel damage during debonding, which is similar to the outcomes of the other studies [11,12,13,24].

Additionally, we found an ARI score of 3 in a group of magnetostrictive scalers, which indicates failure between the bracket and the adhesive. Scribante A. et al. [12] found that ultrasonic instrumentation could be done before the debonding of orthodontic brackets to increase ARI scores, which would likely lower the risk of enamel fracture.

Furthermore, in magnetostrictive scalers, energy from elliptical stroke patterns is converted to vibrations, so all surfaces of the tip are active in debris removal [29]. A study by Yousefimanesh H. et al. [9], applying a magnetostrictive scaler, showed a rougher root surface than a piezoelectric device with the same lateral force. All these factors could have influenced the bracket’s failure.

### 4.3. Load

According to the study’s findings, the force exerted by the tip of the two scalers against the base of the brackets was similar, but could only be obtained from an experienced and trained operator. The second null hypothesis, which was accepted (*p* > 0.05), showed no differences in load between magnetostrictive and piezoelectric scalers. The mean load in this study from both groups (PE and MS) was around 200 g, which is more clinically relevant [30,31].

## 5. Limitations

The buccal tooth surface was a limitation of this study due to the use of teeth from several individuals, which may have influenced the placement of the bracket on the tooth surface. This may affect SBS. Therefore, future studies should concentrate on how to control tooth surface grinding. In addition, more research needs to be done on how other things affect the SBS of orthodontic brackets when ultrasonic devices are used for oral hygiene (such as instrumentation time, power setting, a different type of adhesive or bracket, or a different size and shape of the tip). In addition, further clinical trials are needed.

## 6. Conclusions

In this in vitro study, there were no significant differences in SBS after using 1-min magnetostrictive and piezoelectric ultrasonic scalers around the polypropylene fiber adhesive bracket base. In addition, in all groups, there was no significant difference in ARI scores. The Flash-Free adhesive remained on the enamel, which meant there was a low chance the enamel would be damaged during debonding.

## Figures and Tables

**Table 1:** Statistical descriptions and outcomes of the Bonferroni test for comparing SBS values of the groups (MPa).

Group	N	Mean ± SD	Max	Min	Multiple Comparisons (Bonferroni Test)	
					PE	MS
Control	20	9.65 ± 0.43	12.65	6.70	*p* > 0.05	*p* > 0.05
PE	20	9.45 ± 0.67	15.16	5.98		
MS	20	9.54 ± 0.51	13.67	6.04		

PE, piezoelectric group; MS, magnetostrictive group; N, sample size; SD, standard deviation; Max, maximum value; Min, minimum value.

**Table 2:** The distribution of frequency and percentage and results of the chi-squared test of adhesive remnant index (ARI) scores of each group.

Group	N	ARI Score ^1^				Chi-Squared Test
		0 (%)	1 (%)	2 (%)	3 (%)	
Control	20	0 (0%)	2 (10%)	18 (90%)	0 (0%)	*p* = 0.21
PE	20	1 (5%)	6 (30%)	13 (65%)	0 (0%)	
MS	20	1 (5%)	5 (25%)	12 (60%)	2 (10%)	

^1^ ARI, adhesive remnant index; 0, all adhesive remains on the bracket base; 1, ≥50% of the adhesive remains on the bracket base; 2, <50% of the adhesive remains on the bracket base; and 3, no adhesive remains on the bracket base. PE, piezoelectric group; MS, magnetostrictive group; N, sample size.

**Table 3:** Descriptive statistics and results of the unpaired *t*-test for comparing load values of the groups (*n*).

Group	N	Mean ± SD	Max	Min	Sig. (Unpaired *t*-Test)
PE	20	0.24 ± 0.07	0.38	0.12	*p* = 0.71
MS	20	0.23 ± 0.07	0.39	0.12	

PE, piezoelectric group; MS, magnetostrictive group; N, sample size; SD, standard deviation; Max, maximum value; Min, minimum value; Sig., significance.

## Data Availability

Not applicable.
